# Natural Flavonoid Derivatives Have Pan-Coronavirus Antiviral Activity

**DOI:** 10.3390/microorganisms11020314

**Published:** 2023-01-25

**Authors:** Mattia Mori, Deborah Quaglio, Andrea Calcaterra, Francesca Ghirga, Leonardo Sorrentino, Silvia Cammarone, Matteo Fracella, Alessandra D’Auria, Federica Frasca, Elena Criscuolo, Nicola Clementi, Nicasio Mancini, Bruno Botta, Guido Antonelli, Alessandra Pierangeli, Carolina Scagnolari

**Affiliations:** 1 Department of Biotechnology, Chemistry and Pharmacy, University of Siena, 53100 Siena, Italy; 2 Department of Chemistry and Technologies of Drugs, Sapienza University of Rome, 00185 Rome, Italy; 3 Laboratory of Virology, Department of Molecular Medicine, Sapienza University of Rome, 00185 Rome, Italy; 4 Laboratory of Medical Microbiology and Virology, Vita-Salute San Raffaele University, 20132 Milan, Italy; 5 Laboratory of Medical Microbiology and Virology, IRCCS San Raffaele Hospital, 20132 Milan, Italy

**Keywords:** baicalein, COVID-19, coronavirus

## Abstract

The SARS-CoV-2 protease (3CLpro) is one of the key targets for the development of efficacious drugs for COVID-19 treatment due to its essential role in the life cycle of the virus and exhibits high conservation among coronaviruses. Recent studies have shown that flavonoids, which are small natural molecules, have antiviral activity against coronaviruses (CoVs), including SARS-CoV-2. In this study, we identified the docking sites and binding affinity of several natural compounds, similar to flavonoids, and investigated their inhibitory activity towards 3CLpro enzymatic activity. The selected compounds were then tested in vitro for their cytotoxicity, for antiviral activity against SARS-CoV-2, and the replication of other coronaviruses in different cell lines. Our results showed that Baicalein (100 μg/mL) exerted strong 3CLpro activity inhibition (>90%), whereas Hispidulin and Morin displayed partial inhibition. Moreover, Baicalein, up to 25 μg/mL, hindered >50% of SARS-CoV-2 replication in Vero E6 cultures. Lastly, Baicalein displayed antiviral activity against alphacoronavirus (Feline-CoV) and betacoronavirus (Bovine-CoV and HCoV-OC43) in the cell lines. Our study confirmed the antiviral activity of Baicalein against SARS-CoV-2 and demonstrated clear evidence of its pan-coronaviral activity.

## 1. Introduction

The pandemic threat, caused by the severe acute respiratory syndrome coronavirus 2 (SARS-CoV-2), is still affecting people worldwide and viral variants contribute to the periodic rise in COVID-19 cases. Despite the development of multiple SARS-CoV-2 vaccines and the good degree of immunological protection achieved through vaccination, the mutation of newer variants and the uncertainty about how these detected mutations affect the efficacy of existing anti-spike vaccines have focused research on the development of efficacious drugs for COVID-19 therapeutic intervention.

COVID-19 emerged in late December 2019 [1,2] and subsequently SARS-CoV-2, a positive-strand RNA of the *Coronaviridae* family, was shown to be the cause. Based on numerous studies of the pathogenesis and virulence of coronavirus, distinct viral enzymes, namely 3-chymotrypsin-like cysteine protease (3CLpro), papain-like protease (PLpro), and RNA-dependent RNA polymerase (RDRP), were designated for exploring the potential of anti-SARS-CoV-2 drugs [3,4,5]. In particular, 3CLpro (also known as Mpro) plays a crucial role in viral protein maturation and antagonizes innate immunity [6]. Upon entry into and the uncoating of the SARS-CoV-2 virions, the genomic RNA is translated into two polyproteins, PP1a and PP1ab, which are then cleaved into nonstructural proteins by PL2pro and 3CLpro viral proteases [6]. 3CLpro is responsible for cleaving at eleven sites on the polyproteins processing individual proteins, including the polymerase subunits [6]. Coronavirus 3CLpro remains one of the primary targets for SARS-CoV-2 therapeutics for the following reasons: (i) it has an indispensable role in the proteolytic processing of the viral RNA-polymerase complex, (ii) it is a highly conserved target protein, and (iii) it shares no structural similarity to human proteases [7].

Despite the intense efforts to develop SARS-CoV-2 specific inhibitors [8], only two antiviral agents, authorized by FDA in December 2021, have demonstrated activity in treating mild-to-moderate COVID-19 in patients who are at high risk of severe disease, including molnupiravir, an orally available base analogue that can be incorporated into RNA instead of cytidine triphosphate or uridine triphosphate, leading to mutated RNA products [9] and Paxlovid, a co-packaged combination of nirmatrelvir and ritonavir that acts as a peptidomimetic, covalent, and reversible inhibitor of 3CLpro [10]. The antiviral activity of nirmatrelvir has been reported to not be reduced when treating the SARS-CoV-2 variants Alpha (B.1.1.7), Beta (B.1.351), Gamma (P.1), Delta (B.1.617.2), and Omicron (B.1.1.529) [11], confirming the conservation of the sequence of this enzyme among coronaviruses. Regrettably, cases of patients experiencing a rebound in SARS-CoV-2 viral load and symptoms after completing Paxlovid treatment have been documented [12]. Thus, the development of antiviral therapies directed against viral targets with minimal side effects remains highly urgent.

In the search for additional therapeutic options, several studies conducted in silico, in vitro, and in vivo showed that small natural molecules belonging to polyphenol family [13,14] have the potential to hinder various steps of coronavirus replication. Furthermore, growing understanding of the possible antiviral mechanisms of flavonoids based on previous studies with other coronaviruses, including SARS-CoV-2 [15], and the potential activity of flavonoids to ameliorate post-COVID-19 syndrome through their anti-inflammatory properties [16,17,18] underline the relevance of further exploring the anti-coronavirus activities of these compounds.

To that end, molecular docking was used to screen and determine the docking patterns and binding affinities of compounds with structural similarities to flavonoids, which are the major targets of SARS-CoV-2 3CLpro [19]. Then, an enzymatic assay was employed in order to examine the inhibitory effects of these compounds on 3CLpro. The activity of selected 3CLpro inhibitors in the life cycle of coronavirus was then assessed by evaluating their antiviral activity against SARS-CoV-2 and different alpha (Feline-CoV) and beta coronaviruses (Bovine-CoV and HumanCoV-OC43) in vitro.

## 2. Methods

### 2.1. Study Design

Two Flavonoids (Luteolin and 7,8-hydroxyflavone) have previously been found to be active against 3CLpro [20]. Other compounds with structural analogy were tested through in silico analyses for possible activity against 3CLpro. The compounds that were found to be active in silico were tested through enzymatic assays. Then, the compounds that were found to be active were tested through an in vitro assay for anti-SARS-CoV-2 activity. Lastly, in order to investigate possible anti-pan-coronaviral activity, we evaluated these compounds against Feline (F)-CoV, Bovine (B)-CoV, and Human (H)CoV-OC43.

### 2.2. Chemistry

All the tested compounds (1–12) (Table 1) are known structures belonging to the in-house library of natural products available from the Organic Chemistry Laboratory of the Department of Chemistry and Technology of Drugs of Sapienza University of Rome, Italy [21]. The chemical identity of the compounds was assessed by re-running NMR experiments and proved to be in agreement with the literature data reported below for each compound. The purity of all the compounds, checked by reversed-phase High-Performance Liquid Chromatography (HPLC), was always higher than 95%.

### 2.3. In Silico Screening of Natural Compounds

The crystallographic structure of SARS-CoV-2 3CLpro in complex with a small molecular fragment at 1.95 Å resolution and coded by PDB-ID: 5R81 was selected [50]. In order to relax possible structural constraints, 500 ns of MD simulations were run with AMBER18 using the ff14SB force field for the protein and GAFF for the small molecule. An already validated protocol for classical MD simulations was used [51,52,53]. A representative structure was extracted from the MD trajectory using a cluster analysis, and it was used as a rigid receptor in the subsequent structure-based virtual screening of a proprietary library of natural products [21]. Virtual screening was carried out with the FRED docking program, version 3.3.0.3 (OpenEye Scientific Software) [54,55], in a binding site of 390 Å^3^ that was centered on the binding pose of the crystallographic ligand. For virtual screening purposes, 3D conformers of natural products included in the proprietary library were generated from SMILES using the OMEGA software version 3.1.0.3 (OpenEye Scientific Software) [56], whereas the protonation state at a pH of 7.4 was assigned using QUACPAC version 2.0.0.3 (OpenEye Scientific Software) [57]. Ligand energy minimization was carried out using SZYBKI, version 1.10.0.3 (OpenEye Scientific Software) [58], using the MMFF94S force field.

### 2.4. SARS-CoV-2 3CLpro Assay

In order to examine the compounds with structural analogies to active flavonoids 7,8-Hydroxyflavone and Luteolin for their inhibitory activity against SARS-CoV-2, a preliminary screening using an enzymatic assay [3CLpro, Untagged SARS-CoV-2 Kit, AMSBio (Abingdon, UK) was performed. We tested the compounds at different concentrations and the control inhibitor compounds were incubated with 3CLpro for 30 min, following the manufacturer’s instructions. Subsequently, the substrate of protease was added, and after 4 h of incubation, the fluorescence intensity following the cleavage of the enzyme substrate was measured at an excitation wavelength of 360 nm and an emission wavelength of 460 nm. The “blank” value (fluorescence intensity of the substrate in an assay buffer) was subtracted from each reading and the percentage of inhibition shown by each compound was calculated with respect to untreated 3CLpro activity (0%) and to the control inhibitor compound (100%). All the conditions were performed in triplicate.

### 2.5. Cell Viability Assay to Determine Compounds’ Toxicity

Cell viability was assessed using the Cell Proliferation kit II (XTT) (Roche Diagnostics, Merck, Darmstadt, Germany) as previously described [59]. In this assay, the tetrazolium salt 2,3-bis-(2-methoxy-4-nitro-5-sulfophenyl)-2H-tetrazolium-5-carboxanilide (XTT) was cleaved by viable cells in order to form an orange formazan dye that is then quantified photometrically at 450 nm. Briefly, the cells (4 × 10^5^ cells/mL) were cultured in 96-well plates for 24 h. The culture medium was then replaced by medium containing serial dilutions of the 3CLpro inhibitors, and the cells were incubated for 24, 48, 72, and 96 h. Then, XTT was added to each well and the plates were incubated for 2 h. Optical density was measured at 450 nm (reference wavelength—650 nm) using a Multiskan GO plate reader (Thermo Scientific Instruments, Waltham, MA, USA). For the quantifications, the background levels of media without cultured cells were subtracted.

### 2.6. SARS-CoV-2 In Vitro Assay

Vero E6 (Vero C1008, clone E6—CRL-1586; ATCC) cells were cultured in Dulbecco’s Modified Eagle Medium (DMEM) supplemented with non-essential amino acids (NEAA), penicillin/streptomycin (P/S), Hepes buffer, and 10% (*v*/*v*) Fetal bovine serum (FBS). A clinical isolate of SARS-CoV-2 Omicron BA.5 (hCoV-19/Italy/LOM-UniSR38/2022, GISAID ID: EPI_ISL_15778241) was obtained and propagated in Vero E6 cells, and the viral titer was determined by the 50% tissue culture infective dose (TCID_50_) and plaque assay. All the infection experiments were performed in a biosafety level three (BSL-3) laboratory, as previously described [59].

### 2.7. SARS-CoV-2 Titration

Virus stocks were titrated using the Endpoint Dilutions Assay (EDA, TCID_50_/mL), as described [59]. Briefly, Vero E6 cells (4 × 10^5^ cells/mL) were seeded into 96 wells plates and infected with base 10 dilutions of the virus stock. After 1 h of adsorption at 37 °C, the cell-free virus was removed, and complete medium was added to the cells after a PBS wash. After 72 h, the cells were observed to evaluate CPE. The Reed and Muench method was used to calculate the 50% endpoint using serial dilutions.

### 2.8. Feline-CoV, Bovine-CoV, and HCoV-OC43 In Vitro Assays and Titration

HRT-18 (CCL-244; ATCC) and CRFK (CCL-94; ATCC) cells were cultured in DMEM supplemented with NEAA, P/S, Hepes buffer, and 10% FBS. A clinical isolate of F-CoV (kind gift from Prof. Buonavoglia, University of Bari) was obtained and propagated in CRFK cells, whereas B-CoV (kind gift from Prof. Buonavoglia, University of Bari) was isolated and propagated in HRT-18 cells. HCoV-OC43 was a kind gift from Prof. Baldanti (S. Matteo Hospital, Pavia). A titer of each CoV was measured using RT/Real-Time PCR assays, as previously described [60]. Briefly, the following primers and probes were used [60]: F-CoV (Forward: 5′-GATTTGATTTGGCAATGCTAGATTT-3′; Reverse: 5′-AACAATCACTAGATCCAGACGTTAGCT-3′; Probe: 5′-6FAM/TCCATTGTTGGCTCGTCATAGCGGA/TAM-3′), B-CoV (Forward: 5′-CTGGAAGTTGGTGGAGTT-3′; Reverse: 5′-ATTATCGGCCTAACATACATC-3′; Probe: 5′-6FAM/CCTTCATATCTATACACATCAAGTTGTT/TAM-3′), and HCoV-OC43 (Forward: 5′-AGCAGACCTTCCTGAGCCTTCAAT-3′; Reverse: 5′-AGCAACCAGGCTGATGTCAATACC-3′; Probe: 5′-/6FAM/TGACATTGTCGATCGGGACCCAAGTA/36-TAMSp/-3′.).

### 2.9. Coronaviruses In Vitro Assays

The cells (4 × 10^5^ cells/mL) were seeded into 96-well plates 24 h prior to the experiment. The cells were pretreated with different concentrations of selected 3CLpro inhibitors (1:2 serial dilutions) for 1 h at 37 °C, and were then infected for 1 h with each CoV (0.1 multiplicity of infection, MOI) in the presence of each compound. After a PBS wash to remove cell-free virus particles, 3CLpro-inhibitor-containing medium with 2% FBS was added to the cells and was maintained until the end of the experiment [48 h post-infection (hpi) for F-CoV (CRFK), 72 hpi for SARS-CoV-2 (Vero E6), and 96 hpi for B-CoV (HRT-18) and HCoV-OC43 (HRT-18)]. Then, the SARS-CoV-2 cytopathic effect (CPE) was assessed using a scoring system (0 = uninfected; 0.5 to 2.5 = increasing number/area of plaques; 3 = all the cells are infected), as described [59]. The infection control (score 3) was set as 100% infection and the uninfected cells (score 0) were set as 0% infection. The whole surface of the wells was considered for the analysis (5× magnification). F-CoV, B-CoV, and HCoV-OC43 titers were evaluated through RT/Real-Time PCR and absolute quantifications were calculated using a standard curve [60]. All the conditions were tested in triplicate.

### 2.10. Statistical Analysis

A two-way ANOVA and Dunnett’s multiple comparisons test were performed for cell viability assessment. The CPE observed was normalized to corresponding SARS-CoV-2 infection control. Then, a two-way ANOVA and Tukey’s multiple comparisons test was performed for the evaluation of CPE scoring results (GraphPad Prism 8). The antiviral activity of the compounds tested against F-CoV, B-CoV, and HCoV-OC43 were calculated as the percentage of the reduction of viral RNA copies between untreated infected cells and treated infected cells. A *p*-value below 0.05 was considered significant.

## 3. Results

### 3.1. Virtual Screening of the in-House Natural Products Library

The natural products included in the in-house library (available at Sapienza University of Rome in the research group of Prof. Bruno Botta [21]) were prepared for virtual screening through (i) the generation of 3D conformers, (ii) ionization at a pH of 7.4, and (iii) energy minimization in water solvent with the MMFF94S force field. The crystallographic structure of SARS-CoV-2 3CLpro in complex with the non-covalent inhibitor Z1367324110 (PDB ID: 5R81) was selected as a receptor in structure-based virtual screening [49]. To remove any possible structural bias from protein crystallization, molecular dynamics (MD) simulations were carried out on the 3CL/Z1367324110 crystallographic complex using a previously validated protocol. After 500 ns of unrestrained production of the MD trajectories, the MD frames were clustered using a hierarchical agglomerative algorithm, and the centroid frame of the most populated cluster was selected as representative structure for subsequent structure-based virtual screening.

Virtual screening was performed by molecular docking simulations with a FRED docking program [50,51,52]. Coupling visual inspection of the binding modes with analysis of the Chemgauss4 score led to the selection of 12 small molecules as candidate inhibitors of SARS-CoV-2 3CLpro. All the identified compounds are plant polyphenolic secondary metabolites belonging to the flavonoid family (Table 1). The basic flavonoid structure exists in a diphenylpropane skeleton (C6–C3–C6), namely the flavan nucleus, where two benzene rings (ring A and B) are linked by a three-carbon chain forming a pyran ring C (Table 1). Based on the degree of unsaturation and the oxidation level of ring C, they can be divided in different subclasses: Morin (1), Quercetin (2), Alnusin (3), Isokaempferide (4), and Galangin (5) are flavonols featuring a 3-hydroxyflavone backbone structure; Steppogenin (6), Sakuranetin (7), and Isosakuranetin (8) are flavanones featuring a flavan backbone; Baicalein (9), Hispidulin (10), and Chrysin (11) are flavones; and Taxifolin (12), which features a 3-hydroxyflavanone backbone, is a flavanonol.

### 3.2. SARS-CoV-2 3CLpro Assay

We evaluated the inhibitory activity of compounds 1–12 against the 3CLpro of SARS-CoV-2 in on-plate enzymatic assays at the following concentrations: 100 µg/mL and serial 1:2 dilutions reaching a final concentration of 0.78 µg/mL. We observed substantial inhibitory activity of Baicalein (94.72%) at the concentration of 100 µg/mL (Table 2), whereas for Hispidulin and Morin, partial inhibition of the protease activity was observed at 100 µg/mL (55.62% and 53.64%, respectively) (Table 2). Isokaempferide exhibited a detectable effect on 3CLpro when used at concentrations ranging from 100 µg/mL to 25 µg/mL, leading to a percentage of reduction of its activity of 49.98%, 40.06%, and 19.69%, respectively. We also examined the inhibitor effects of Luteolin and 7,8-dihydroxy-flavone, for which antiviral activity has been previously reported [20,61], finding low activity against 3CLpro at 100 µg/mL (38.12% and 39.80%, respectively), and undetectable activity at 25 µg/mL (Table 2). By contrast, no activity against 3CLpro was observed for Taxifolin, Steppogenin, Quercetin, Chrysin, Sakuranetin, Alnusin, Galangin, and Isosakuranetin when used at concentrations ranging from 100 µg/mL to 6.125 µg/mL (data not shown).

Given the Morin, Baicalein, Hispidulin, and Isokaempferide had detectable activity against SARS-CoV-2 3CLpro, we investigated the efficacy of these compounds in the impairment of SARS-CoV-2 replication on Vero E6 cells with an in vitro assay.

### 3.3. Effect of the 3CLpro Inhibitors on VERO E6 Viability

**Figure 1:**
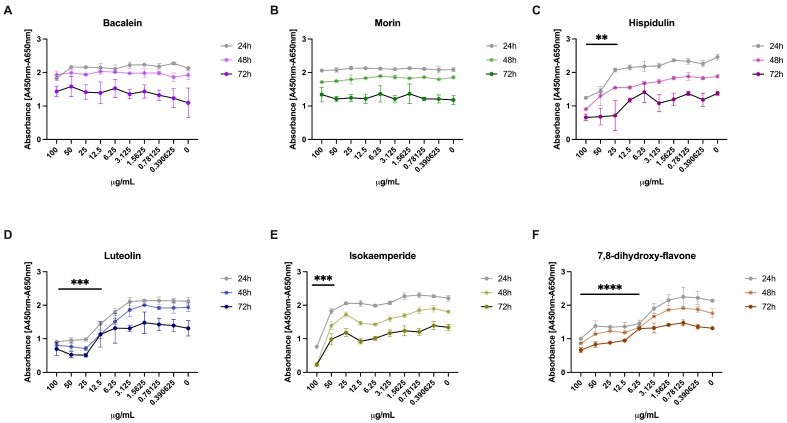
Cell viability analysis. Different concentrations (100–0.4 μg/mL) of the selected 3CLpro inhibitors (**A**–**F**) were tested in order to assess their toxicity on Vero E6. Absorbance was reported as mean values ± SD, ** *p* < 0.01, *** *p* < 0.001, and **** *p* < 0.0001.

Vero E6 cells were tested with serial dilutions of Morin, Baicalein, Hispidulin, Isokaempferide, Luteolin, and 7,8-dihydroxyflavone in order to assess their cytotoxicity. The results showed no difference between the treated and untreated cells when Baicalein and Morin were used, even after testing higher concentrations for 72 h (Figure 1A,B). Instead, the other compounds induced some degree of cytotoxicity. In detail, Hispidulin (Figure 1C) was found to be toxic when used at 50 μg/mL after 24 and 48 h (*p* < 0.0001 and *p* < 0.01, respectively) and at 25 μg/mL after 72 h of treatment (*p* < 0.01). Luteolin (Figure 1D) impaired cell viability starting from 12.5 μg/mL at each considered time point (*p* < 0.001 after 48 and 72 h, *p* < 0.01 after 24 h), as well as Isokaempferide (Figure 1E) starting from 50 μg/mL (*p* < 0.001 at all the time points), and 7,8-dihydroxy-flavone starting from 6.25 μg/mL (*p* < 0.0001) at all the time points.

### 3.4. Antiviral Activity of 3CLpro Inhibitors against SARS-CoV-2

**Figure 2:**
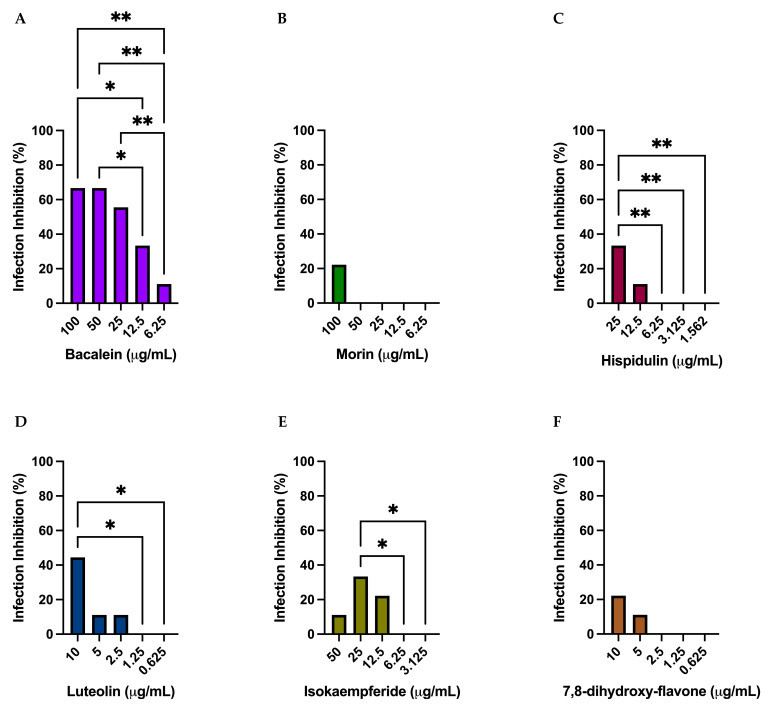
(**A**–**F**) Antiviral activity of selected 3CLpro inhibitors. The inhibition of Omicron BA.5 infection was tested using different concentrations of the selected compounds and was assessed at 72 hpi. Mean values are reported for all the experimental replicates. Standard deviations were less than 10% of the mean values. An ordinary one-way ANOVA with a Tukey’s multiple comparisons test, with a single pooled variance, was performed. * *p* < 0.05 and ** *p* < 0.01.

Vero E6 cells were treated with serial dilutions of the selected compounds, relying on cell viability data to determine the highest concentrations to test: 100 μg/mL for Baicalein and Morin (Figure 2A,B), 25 μg/mL for Hispidulin (Figure 2C), 10 μg/mL for Luteolin and 7,8-dihydroxy-flavone (Figure 2D,F), and 50 μg/mL for Isokaempferide (Figure 2E). The inhibitors were added one hour before inoculation with the SARS-CoV-2 Omicron BA.5 variant (0.1 Multiplicity of infection, MOI), and were monitored for cytopathic effects at 72 hpi. The results showed that no compound, at any concentration, could fully protect the cells from infection with the virus. Only the treatment with Baicalein hindered virus replication above 50%, starting from a concentration of 25 μg/mL.

### 3.5. Effect of the 3CLpro Inhibitors on CRFK and HRT-18 Viability and Pan-Coronaviral Activity

In order to investigate possible anti-pan-coronaviral activity, we tested the efficacy of Baicalein and Luteolin against Feline-CoV (F-CoV), Bovine-CoV (B-CoV) and human-CoV (HCoV) OC43 replication.

CRFK and HRT-18 were tested with serial dilutions of Baicalein and Luteolin in order to assess their cytotoxicity. The highest concentrations with no toxic activity were 50 μg/mL for Baicalein and 3.125 μg/mL for Luteolin in both the cell lines. In order to test pan-coronavirus antiviral activity, Baicalein and Luteolin were added 1 h before inoculation with F-CoV on CRFK cells, B-CoV, and HCoV-OC43 on HRT-18 cells (0.1, Multiplicity of infection, MOI) and were monitored 48 hpi (F-CoV) and 96 hpi (B-CoV and HCoV-OC43). Only Baicalein showed antiviral activity (Table 3); it inhibited F-CoV and B-CoV at 50 μg/mL and at lower concentrations (up to 12.5 μg/mL) inhibited HCoV-OC43 replication (Table 3).

### 3.6. Predicted Binding Mode of Most Effective SARS-CoV-2 3CLpro Inhibitors

**Figure 3:**
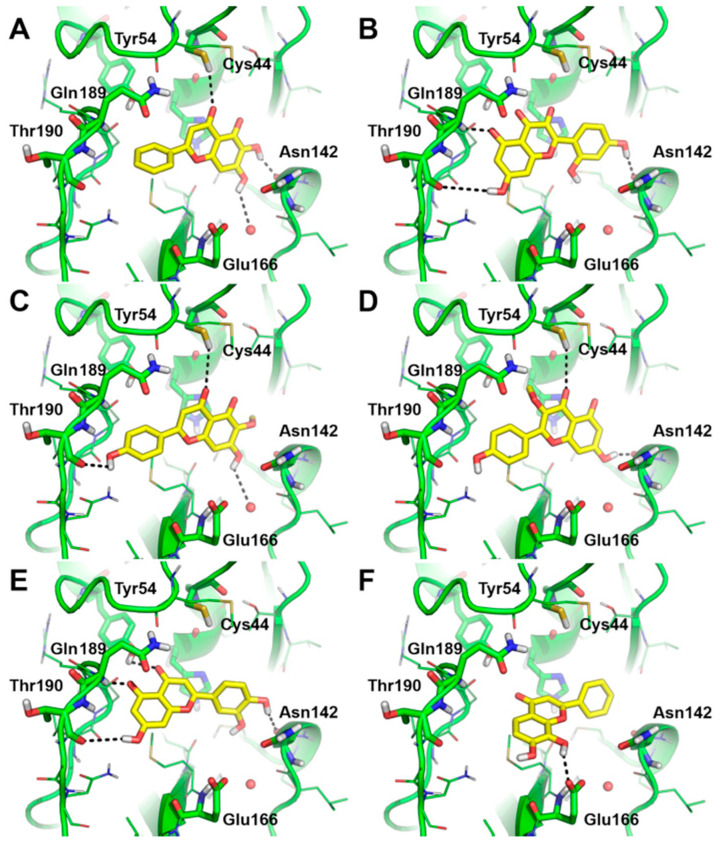
Putative binding modes of the bioactive natural products investigated in this work within the catalytic site of SARS-CoV-2 3CLpro, as predicted by molecular docking simulations. (**A**) Baicalein; (**B**) Morin; (**C**) Hispidulin; (**D**) Isokaempferide; (**E**) Luteolin; and (**F**) 7,8-dihydroxyflavone. The protein is shown as a green cartoon and lines (only residues within 6 Å from the ligands are shown, the others are omitted). The ligand polar contacts are highlighted by black dashed lines. The residues contacted by the ligands are labeled. Natural products are shown as yellow sticks, and non-polar H atoms are omitted.

The possible binding mode of the bioactive compounds discussed above within the catalytic site of SARS-CoV-2 3CLpro was investigated by molecular docking simulations. Since all the docked compounds are members of the flavone family, although they bear different substitution patterns, it is not surprising that they bind within the catalytic site of SARS-CoV-2 3CLpro in a highly consistent manner to each other, as well as to the docking-based binding mode of other flavonoids from the literature [62]. Specifically, Morin, Luteolin, and the 7,8-dihydroxyflavone bind with opposite orientations with respect to Baicalein, Hispidulin, and Isokaempferide. The latter of these have the polyhydroxylated ring A of the flavone scaffold projected to the inner core of 3CLpro that is in the proximity of Asn142 (Figure 3). Besides stacking to the side chain of His41, the natural compounds establish H-bond interactions with the backbone of Asn142 (Baicalein, Isokaempferide, and Morin), with the phenolic side chain of Try54 (Luteolin), with the backbone of Gln189 (Morin and Luteolin), with the backbone of Thr190 (Morin, Hispidulin, and Luteolin), and with the side chain of Cys44. Only 7,8-dihydroxyflavone establishes an H-bond interaction with the side chain of Glu166 (Figure 3). Notably, Baicalein and Hispidulin are able to H-bond a water molecule, which was identified by the MD simulations and is found in X-ray structures (Figure 3A,C).

The predicted binding mode supports the inhibition of SARS-CoV-2 3CLpro by these natural compounds, as was confirmed by the experimental studies.

## 4. Discussion

The COVID-19 pandemic has revealed the urgent need for novel antiviral drugs. Flavonoids have been largely studied as possible inhibitors of 3CLpro and several studies found them to be active against SARS-CoV and MERS-CoV proteases [19]. It has previously been shown that several of these compounds can impair HCoV-229E replication in vitro [63]. The antiviral activity of Quercetin was previously observed in vitro against HSV-1 [64] and during in vitro and in vivo Rhinovirus infection [65], and its derivates also show antiviral activity against Respiratory Syncytial Virus (RSV) [66] and Influenza A H1N1 virus replication in cell cultures [67]. During the COVID-19 pandemic, many flavonoids were examined against SARS-CoV-2 3CLpro. In particular, it has been observed that myricetin covalently binds to the Cys300 and Cys41 of 3CLpro [68].

In this study, we identified in silico flavonoid derivatives and subsequently evaluated a library of flavonoid derivatives for their inhibitory activity on 3CLpro. Firstly, we identified Morin, Baicalein, Hispidulin, Taxifolin, Steppogenin, Quercetin, Chrysin, Sakuranetin, Alnusin, Galangin, Isosakuranetin, and Isokaempferide through in silico analyses.

Then, we tested these flavonoids in a 3CLpro enzymatic assay, finding four of them, Morin, Baicalein, Hispidulin, and Isokaempferide, to be active against 3CLpro. Baicalein, the most promising compound, inhibited 3CLpro activity above 90%, and Morin, Hispidulin, and Isokaempferide were partially active against SARS-CoV-2 3CLpro. The antiviral activity of Morin was observed against the *Herpesviridae* family [69], whereas Hispidulin has been associated with Influenza A H1N1 neuraminidase inhibition [70].

In order to assess their anti-coronaviral activity, we tested these compounds against SARS-CoV-2 replication with an in vitro assay, but only Baicalein had more than 50% activity against virus replication at concentrations that were non-toxic for Vero E6 cells. In agreement, Seri et al. [4] found Baicalin, but also Herbacetin and Pectolinarin, to be effective against SARS-CoV-2. Other studies have reported in vitro Baicalein to have antiviral activity against SARS-CoV-2 [71]. The anti-SARS-COV-2 properties of Baicalein were, in part, explained through the observation of in silico interactions between this compound and 3CLpro [15,72]. Furthermore, a crystallographic picture of this molecular interaction was obtained with SARS-CoV-1 3CLpro [73], which has 96% similarity with SARS-CoV-2 [74].

Since the emergence of SARS-CoV-1 in 2002 [75], MERS-CoV in 2012 [76], and SARS-CoV-2 in 2019 [77], research has uncovered many details of the life cycle of coronavirus and its pathogenesis; however, there are currently no approved drugs with anti-pancoronaviral activity.

Thus, we extended our observations on the anti-SARS-CoV-2 activity of Baicalein, testing its inhibitory potency against additional members of the *Coronaviridae* family. To our knowledge, for the first time we found that this compound is highly active against F-CoV, B-CoV, and HCoV-OC43 replication. F-CoV [78,79] and HCoV-OC43 [80] have been previously used as SARS-CoV-2 surrogates in order to test the virucidal and antiviral activity of several compounds.

Considering that F-CoV belongs to the alpha coronavirus genus, Baicalein could be active against the human alpha Cov species, i.e., HCoV-229E which usually infects the upper respiratory tract, but can cause more severe diseases in newborns and in immunocompromised individuals [81], and HCoV-NL63 which causes bronchiolitis in infants [82] and pneumonia.

Furthermore, from a translation point of view, the activity of Baicalin against HCoV-OC43 is relevant. Indeed, this HCoV causes very mild disease in immunocompetent adults [81], but it is a threat for newborns (0–1 year) causing bronchiolitis [83].

The broad-spectrum of the anti-pancoronaviral action of Baicalein against F-CoV, B-CoV, and HCoV-OC43 could be explained by the fact that 3CLpro is highly conserved within the *Coronaviridae* family [74]. Of note, the anti-inflammatory activity of flavonoids has also been shown, modulating the expression of inflammatory markers, such as IL1-B and Tumor Necrosis Factor Alpha (TNF-a) [84], and it can modulate the NLRP3 inflammasome [85,86]. Interestingly, IL1-B and TNF-a blood levels were associated with post-COVID-19 illness [87] and the use of flavonoid-derived compounds could mitigate post-COVID-19 symptoms.

In this context, Baicalein has been shown to interact with the NLRP3 inflammasome [86] and with Nf-kB through Nrf2 activation [88].

The limitations of this study include a lack of other HCoV models, including low (HCoV-NL63 and HKU1) and high (MERS-CoV) human pathogenetic coronavirus, but also additional animal coronaviruses (bat coronaviruses) in order to confirm the anti-pan-coronaviral activity of Baicalein. The availability of a compound with proven activity against emerging coronaviruses would represent great value as coronaviruses infecting animals still represent a threat. Indeed, a novel CoV infecting pigs, named swine acute diarrhea syndrome (SADS)-CoV that recently caused an outbreak in China [89], is capable of infectibg human derived cells [90], suggesting that it might also be capable of jumping to humans.

Moreover, several compounds were found to be toxic for the cells and were tested against SARS-CoV-2 in low concentrations. The latter could have underestimated the evaluation of the antiviral potency of these molecules and indicates the need to improve the synthesis processes and test new Baicalein-derived compounds against coronaviruses.

In conclusion, our study confirmed the in vitro antiviral activity of Baicalein against SARS-CoV-2 replication, but also demonstrated clear evidence of the anti-pan-coronaviral activity exerted by this compound. Given that Baicalein and the other bioactive compounds highlighted in this work belong to a very large family of natural compounds, this work paves the way for the exploration of naturally occurring flavonoids in the identification of additional and effective 3CLpro inhibitors of SARS-CoV-2 replication. Starting from our results, future investigations are warranted in order to elucidate the possible activity of Baicalein and its derivatives against other steps of the coronavirus life cycle. Furthermore, we underline the importance of using an experimental approach combining in silico and in vitro analyses in order to uncover novel antiviral drugs that are suitable for treating SARS-CoV-2 in this current pandemic or for the next emerging viral pathogen.

## Figures and Tables

**Table 1:** Flavonoid family compounds.

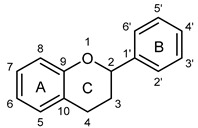 Basic Flavonoid Skeleton						
Mol.	Common Name	Chemical Structure	M.W.	Molecular Formula	Source	Reference
Flavanones						
**1**	Morin	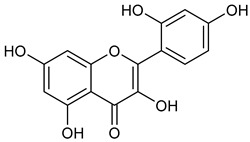	302.24	C_15_H_10_O_7_	Moriaceae family	[22]
**2**	Quercetin	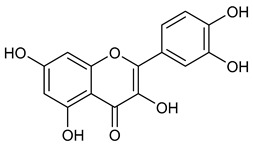	302.24	C_15_H_10_O_7_	*Ginkgo biloba* (Ginkgoaceae family) *Hypericum perforatum* (Hypericaceae family) *Sambucus canadensis* (Adoxaceae family)	[23,24]
**3**	Alnusin	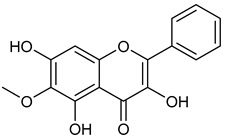	300.27	C_16_H_12_O_6_	*Xerochrysum viscosum* (Asteraceae family) *Alnus sieboldiana* (Betulaceae family)	[25]
**4**	Isokaempferide	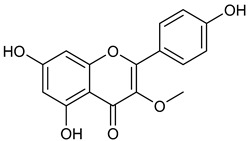	300.27	C_16_H_12_O_6_	*Amburana cearensis* (Fabacee family)	[26]
**5**	Galangin	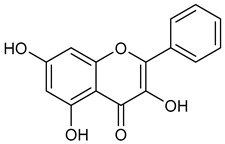	270.24	C_15_H_10_O_5_	*Alpinia officinarum* (Zingiberaceae family) *Helichrysum aureonitens* (Asteraceae family) *Alpinia galanga* (Zingiberaceae family)	[27,28,29]
Flavanones						
**6**	Steppogenin	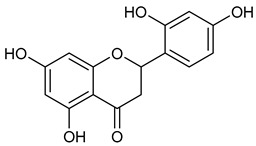	288.26	C_15_H_12_O_6_	*Euphorbia nicaeensis* (Euforbiacee family) *Maclura tricuspidata* (Moraceae family)	[30]
**7**	Sakuranetin	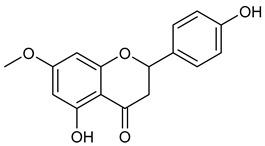	286.28	C_16_H_14_O_5_	*Polymnia fruticosa* (Araliaceae family)	[31]
**8**	Isosakuranetin	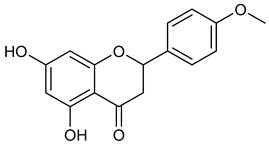	286.28	C_16_H_14_O_5_	*Monarda didyma* (Lamiaceae family)	[32]
Flavones						
**9**	Baicalein	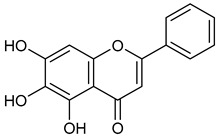	270.24	C_15_H_10_O_5_	*Scutellaria baicalensis* (Lamiaceae family)	[33]
**10**	Hispidulin	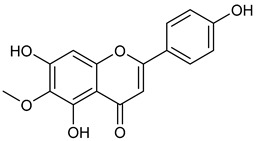	300.27	C_16_H_12_O_6_	*Crossostephium chinense*, *Grindelia argentina* and *Saussurea involucrate* (Asteraceae family) *Arrabidaea chica* (Bignoniaceae Family)	[34,35]
**11**	Chrysin	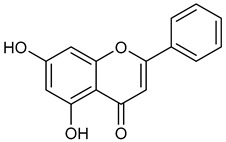	254.24	C_15_H_10_O_4_	*Passiflora caerulea* and *Passiflora incarnata* (Passifloraceae family) *Oroxylum indicum* (Bignoniaceae family)	[36]
Flavanonol						
**12**	Taxifolin	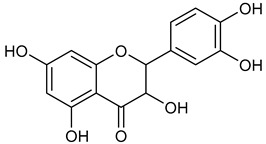	304.25	C_15_H_12_O_7_	*Pinus roxburghii*, *Cedrus deodara* (Pinaceae family)	[37]

Compound 1 (Morin or 2-(2,4-dihydroxyphenyl)-3,5,7-trihydroxy-4*H*-chromen-4-one) showed NMR spectra identical to those reported in the literature [38]. Compound 2 (Quercetin or 2-(3,4-dihydroxyphenyl)-3,5,7-trihydroxy-4*H*-chromen-4-one) showed NMR spectra identical to those reported in the literature [39]. Compound 3 (Alnusin or 3,5,7-trihydroxy-6-methoxy-2-phenyl-4*H*-chromen-4-one) showed NMR spectra identical to those reported in the literature [40]. Compound 4 (Isokaempferide or 5,7-dihydroxy-2-(4-hydroxyphenyl)-3-methoxy-4*H*-chromen-4-one) showed NMR spectra identical to those reported in the literature [41]. Compound 5 (Galangin or 3,5,7-trihydroxy-2-phenyl-4*H*-chromen-4-one) showed NMR spectra identical to those reported in the literature [42]. Compounc 6 (Steppogenin or 2-(2,4-dihydroxyphenyl)-5,7-dihydroxychroman-4-one) showed NMR spectra identical to those reported in the literature [43]. Compound 7 (Sakuranetin or 5-hydroxy-2-(4-hydroxyphenyl)-7-methoxychroman-4-one) showed NMR spectra identical to those reported in the literature [44]. Compound 8 (Isosakuranetin or 5,7-dihydroxy-2-(4-methoxyphenyl)chroman-4-one) showed NMR spectra identical to those reported in the literature [45]. Compound 9 (Baicalein or 5,6,7-trihydroxy-2-phenyl-4*H*-chromen-4-one) showed NMR spectra identical to those reported in the literature [46]. Compound 10 (Hispidulin or 5,7-dihydroxy-2-(4-hydroxyphenyl)-6-methoxy-4*H*-chromen-4-one) showed NMR spectra identical to those reported in the literature [47]. Compound 11 (Chrysin or 5,7-dihydroxy-2-phenyl-4*H*-chromen-4-one) showed NMR spectra identical to those reported in the literature [48]. Compound 12 (Taxifolin or 2-(3,4-dihydroxyphenyl)-3,5,7-trihydroxychroman-4-one) showed NMR spectra identical to those reported in the literature [49].

**Table 2:** Enzymatic anti-3CLpro activity of selected compounds.

Compound	Molecular Model	Concentrations Tested (µg/mL)	Percentage of Inhibition of 3CLpro
Morin	Docking	100.00	53.64 (33.85)
		50.00	18.64 (4.36)
		25.00	<10
		12.5	<10
Baicalein	Docking	100.00	94.72 (9.11)
		50.00	72.14 (7.75)
		25.00	25.57 (6.67)
		12.5	17.28 (1.11)
Hispidulin	Docking	100.00	55.62 (17.04)
		50.00	29.76 (12.56)
		25.00	<10
		12.5	<10
Luteolin	Docking	100.00	38.12 (2.32)
		50.00	30.88 (3.53)
		25.00	<10
		12.5	<10
7,8-Dihydroxy-flavone	Docking	100.00	39.8 (0.31)
		50.00	34.54 (0.13)
		25.00	<10
		12.5	<10
Isokaempferide	Cluster	100.00	49.98 (22.38)
		50.00	40.06 (27.12)
		25.00	19.69 (13.71)
		12.5	<10

Data of the inhibition percentage of 3CLpro activity are shown as a mean of the three experiments (standard deviations).

**Table 3:** Pan-coronavirus antiviral activity of Baicalein.

Compound	Concentrations Tested (µg/mL)	Antiviral Activity against F-CoV	Antiviral Activity against B-CoV	Antiviral Activity against OC43
Baicalein	100	TX	TX	TX
	50	66.73	99.60	99.99
	25	<50	89.85	99.99
	12.5	<50	<50	99.98
	6.25	<50	<50	<50
	3.125	<50	<50	<50
	1.56	<50	<50	<50
	0.78	<50	<50	<50

Data are expressed as a percentage. Antiviral activity is expressed as 1-(1/10LRV) * 100. All the experiments were performed in triplicate. Standard deviations were less than 10% of the mean values. TX: Toxic; LRV: Log Reduction Value.

## Data Availability

The datasets analysed during this study are available from the corresponding author on reasonable request.
